# Multi-locus characterization and phylogenetic inference of *Leishmania* spp. in snakes from Northwest China

**DOI:** 10.1371/journal.pone.0210681

**Published:** 2019-04-25

**Authors:** Han Chen, Jiao Li, Junrong Zhang, Xianguang Guo, Jinlong Liu, Jinlei He, Qi Song, Jianhui Zhang, Minli Chen, Zhiwan Zheng, Dali Chen, Jianping Chen

**Affiliations:** 1 Department of Parasitology, West China School of Basic Medical Sciences and Forensic Medicine, Sichuan University, Chengdu, Sichuan, China; 2 Chengdu Institute of Biology, Chinese Academy of Sciences, Chengdu, Sichuan, China; 3 University of Chinese Academy of Sciences, Beijing, China; National Cheng Kung University, TAIWAN

## Abstract

**Background:**

Leishmaniasis caused by protozoan parasite *Leishmania* is a neglected disease which is endemic in the northwest of China. Reptiles were considered to be the potential reservoir hosts for mammalian Leishmaniasis, and *Leishmania* had been detected in lizards from the epidemic area in the northwest of China. To date, few studies are focused on the natural infection of snakes with *Leishmania*.

**Methods:**

In this study, 15 snakes captured from 10 endemic foci in the northwest of China were detected *Leishmania* spp. on the base of mitochondrial *cytochrome b*, *heat shock protein 70* gene and *ribosomal internal transcribed spacer 1* regions, and identified with phylogenetic and network analyses.

**Result:**

In total, *Leishmania* gene was found in 7 snakes. The phylogenetic inference trees and network analysis suggests that the species identification was confirmed as *Leishmania donovani*, *L*. *turanica* and *L*. (*Sauroleishmania*) sp.

**Conclusion:**

Our work is the first time to investigate the natural *Leishmania* spp. infection of snakes in the northwest of China. Mammalian *Leishmania* (*L*. *donovani and L*. *turanica*) was discovered in snakes and the reptilian *Leishmania* (*Sauroleishmania* sp.) was closely related to the clinical strains both prompt the importance of snakes in the disease cycle. To indicate the epidemiological involvement of snakes, a wide sample size in epidemic area and the pathogenic features of reptilian *Leishmania* promastigotes are recommended in the future research.

## Introduction

Leishmaniasis is a neglected disease caused by infection with flagellate protozoan parasite *Leishmania* of the family Trypanosomatidae. There are three main forms of leishmaniasis: visceral or kala-azar (VL), cutaneous (CL) and mucocutaneous (MCL). About 20 *Leishmania* species known to be infective to humans are transmitted by the bite of infected sand flies of which about 166 species were proven or probable vectors worldwide [1]. An estimated 2 million new cases and 50 000 deaths occur in over 98 countries annually [2]. In China, which is one of 14 high-burden countries of VL [3], there exists three epidemiological types: anthroponotic VL (AVL), mountain-type zoonotic VL (MT-ZVL), and desert-type ZVL (DT-ZVL) [4]. Information on the epidemiology including the vector and animal reservoir hosts of the disease is important to understand the disease and its control.

In view of the topography in the northwest of China, DT-ZVL is found largely in oases and deserts including the southern and eastern of Xinjiang Uygur Autonomous Region, the western part of the Inner Mongolia Autonomous Region, and northern Gansu province [5]. Surveillance of the disease reflects that DT-ZVL is persistent with sporadic outbreaks and still not under control now [5]. According to the data from Disease Reporting Information System of China CDC, there were two outbreaks of VL occurred in Xinjiang Uygur autonomous region, of which the highest incidence rate was in Kashgar Prefecture [6,7]. Sand flies (Phlebotominae) were recognized as the transmission vector in the traditional sense, while ticks (Ixodoidea) had been reported to be the vector in the transmission of canine VL [8–11]. In addition to humans, there are various kinds of reservoir hosts, mainly mammals such as canids, hyraxes and rodents, which could be the source of infection. Because transmission occurs in a complex biological system involving the human host, parasite, vectors and in some cases an animal reservoir host, the control of Leishmaniasis is relatively complex. As for the desert in the northwest of China, there is no consistent agreement regarding dissemination of the actors playing the key roles in leishmaniasis.

Moreover, reptiles, mainly lizards, were found harboring *Leishmania* parasites with controversies concerning their role in spreading the disease [12]. Blood cells of lizards containing amastigotes were first found by Chatton and Blanc in *Tarentola mauritanica* from southern Tunisia [13] and later several cases were reported from the same lizard species [14]. In 1920, Wenyon provisionally named the leptomonad flagellates in a gecko as *Leishmania tarentolae* [15]. Killick-Kendrick recognized *Sauroleishmania* as a separate genus for the leishmanial parasites of reptiles in 1986 [16], later DNA sequence-based phylogenies had clearly placed *Sauroleishmania* within the *Leishmania* genus as a secondarily derived development from the mammalian species [17–19]. Most studies were focused on the infection of *Leishmania* parasites in lizards, but Belova and Bogdanov were able to culture promastigotes from the blood of five species of snakes in the Turkmen S.S.R. in 1969 [16,20].

In reality, the detection of reptilian *Leishmania* is rare in China. Zhang et al. was the first team to detect *Leishmania* via molecular methods from lizards captured in the northwest of China [21]. Interestingly, besides reptilian *Leishmania*, mammalian *Leishmania* species (*L*. *donovani* and *L*. *tropica*) were also isolated in their study. There are few studies reporting that mammalian *Leishmania* can parasitize in reptilians. In addition, *Sauroleishmania* was once considered not to infect humans or other mammals [1,22], however, *L*. *adleri* was found to infect rodents [23] and *L*. *tarentolae* could invade human macrophages in later studies [24].

Similarly, we employed the highly sensitive polymerase chain reaction (PCR) to detect the presence of *Leishmania* spp. in snakes. The sequences of various genetic markers have been used successfully to infer the phylogenetic relationships within *Leishmania* including the sequences of DNA polymerase α [17], RNA polymerase II [17], 7SL RNA [25–27], ribosomal internal transcribed spacer [28,29], mitochondrial *cytochrome b* gene [30,31], *heat shock protein 70* gene [32,33] and the *N-acetylglucosamine-1-phosphate transferase* gene [34]. Therefore, this study was based on three genetic loci, i.e., *cytochrome b* (*cyt b*), *heat shock protein 70* (*Hsp70*), and *internal transcribed spacer 1* (*ITS1*) region to genetically characterize *Leishmania* spp. detected in 15 snakes, and conduct tree-based species delimitation to infer the phylogenetic positions by comparison with some representative *Leishmania* sequences retrieved from GenBank. It was the first time to survey infected snakes of *Leishmania* in the northwest of China and to conclude on the role of snakes in the transmission of human leishmaniasis.

## Materials and methods

### Study area and sampling

15 snakes identified as 3 species by morphological characters were captured alive by hand and snake hooks at 10 sites across the endemic foci of VL in the northwest of China (Table 1). These sites are located in arid desert areas with the altitude ranging from 537 to 1222 m above sea level. Franchini [35,36] had observed rare amastigotes in the liver of lizards, and Shortt & Swaminath [37] cultured a strain *Leishmania* on NNN (Novy-MacNeal-Nicolle) medium from the liver of lizards. Consequently, a part of the liver was taken from the abdominal cavity of each snake to detect the presence of *Leishmania* via PCR. In total, 15 liver specimens were collected in Eppendorf tube and stored at -20°.

**Table 1:** List of sampling localities, snake species and sample size in this study.

Site Label	Locality	Species	Sample size
P1	Shawan county, Tarbagatay Prefecture, Xinjiang	*Psammophis lineolatus*	1
P2	Tekes County, Ili Kazak Autonomous Prefecture, Xinjiang	*Gloydius halys*	1
P3	Tekes County, Ili Kazak Autonomous Prefecture, Xinjiang	*Natrix tessellate*	1
P4	Yiwu County, Hami, Xinjiang	*Psammophis lineolatus*	1
P5	Yizhou Area, Hami, Xinjiang	*Psammophis lineolatus*	5
P6	Dunhuang, Jiuquan City, Gansu Province	*Psammophis lineolatus*	1
P7	Heshuo, Bayingol Mongolian Autonomous Prefecture, Xinjiang	*Psammophis lineolatus*	2
P8	Makit County, Kashgar Prefecture, Xinjiang	*Psammophis lineolatus*	2
P9	Yopurga County, Kashgar Prefecture, Xinjiang	*Psammophis lineolatus*	1
P10	Yengisar County, Kashgar Prefecture, Xinjiang	*Psammophis lineolatus*	2
total			15

All surgery was performed under sodium pentobarbital anesthesia, and all efforts were made to minimize suffering. The protocol was approved by the ethics committee of Sichuan University (Protocol Number: K2018056).

### DNA extraction, amplification, cloning and sequencing protocols

Total genomic DNA was extracted from liver tissues using the commercial kit, TIANamp Genomic DNA Kit (TIANGEN Bio, Beijing, China) following the protocols of the manufacturer. PCR primers specific for the *Leishmania* synthesized by Tsingke Biological Technology Co., Ltd (Chengdu, China) were used to amplify *cyt b* [38], *Hsp70* [39] and *ITS1* [40] gene fragments by PrimeSTAR Max DNA polymerase (TaKaRa Bio, Shiga, Japan) according to the manufacturer’s instruction. We picked the Chinese *L*. (*Sauroleishmania*) sp. strain MHOM/CN/90/SC10H2 as the positive control while preparing the mixture, and the negative control was treated without template DNA. The PCR products were purified by excision of the band from agarose gel using the Universal DNA Purification Kit (TIANGEN Bio, Beijing, China), and ligated into a pGEM-T vector (Promega Co., Madison, USA) with added the poly (A) tails. After transformation of *Escherichia coli* DH5α competent cells with the ligation product and X-gal blue-white screening, 8 positive colonies were selected by PCR using the plasmid primers T7 and SP6 and grown in LB/ampicillin medium. DNA was extracted and sequenced at Tsingke Biological Technology Co., Ltd (Chengdu, China). The chromatograms were validated and assembled in BioEdit v7.2.6 [41].

### Sequence alignment and phylogenetic analyses

GenBank searches were performed to initially identify the species of the original DNA sequences using BLASTn (https://blast.ncbi.nlm.nih.gov/Blast.cgi). And all the nucleotide sequences generated in this study have been submitted to the GenBank database. The sequences were multiple-aligned with a set of *Leishmania* strains of each locus examined in this study retrieved from the GenBank (see S1 Table in Supporting information) using ClustalW of MEGA (Molecular Evolutionary Genetic Analysis v7.0.26 [42]) with its default option and refined manually. The alignments were further trimmed to exclude regions with missing data and then distinct haplotypes were defined by DAMBE v7.0.1 [43,44]. The evolutionary history was inferred by phylogenetic tree construction using Bayesian inference (BI). Gaps were treated as missing data and each haplotype was treated as a taxon in the analyses. The program PartitionFinder v2.1.1 [45] was used to select the most appropriated substitution model of all phylogenetic analyses. Bayesian analyses were carried out using the program MrBayes v3.2 [46] which the trees were started randomly. 2 parallel sets of 4 simultaneous Monte Carlo Markov chains (3 hot and 1 cold) were run for 10,000,000 generations until convergence was reached (stopval = 0.01) and the trees were sampled for every 1000 generations. Temperature heating parameter was set to 0.2 (temp = 0.2) and burn-in was set to 25% (burninfrac = 0.25). The results of Bayesian analyses were visualized in FigTree v1.4.3 (available at http://tree.bio.ed.ac.uk/software/figtree/).

### Network reconstructions

Due to the limit of bifurcating tree to represent intraspecific gene evolution, haplotype networks may more effectively portray the relationships among haplotypes within species. The phylogenetic networks were constructed by using the Median Joining (MJ) network reconstruction method in NETWORK v5.0.0.3 (available at http://www.fluxus-engineering.com/sharenet.htm) [47,48].

## Results

### *Leishmania* infection of the captured snakes in the study area

**Fig 1:**
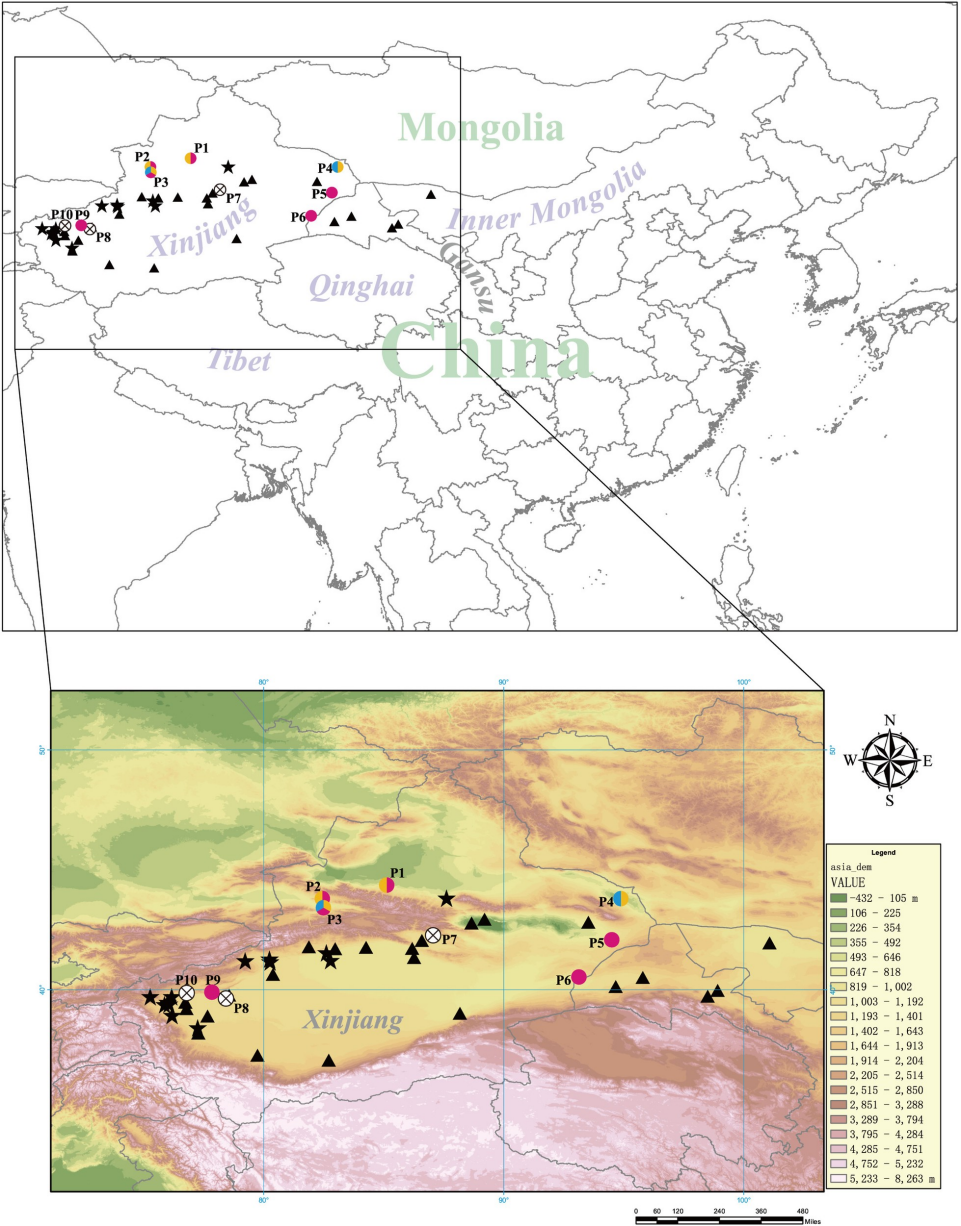
Sampling sites of snakes in Northwest China, along with the current foci of endemicity of anthroponotic visceral leishmaniasis (AVL) and desert-type zoonotic visceral leishmaniasis (DT-ZVL) in China. The map image of countries with boundary lines and the topographic map layer of areas were downloaded from ArcGIS online via ArcMap program (http://www.esri.com). The site numbers P1-P10 correspond to those in Table 1. Solid five-pointed star (★) represents the endemic focus of AVL and solid triangle (▲) represents the endemic focus of DT-ZVL [4,5]. The species of *Leishmania* detected from the samples are shown for different colors (yellow: *L*. *donovani* complex, blue: *L*. *turanica*, pink: *L*. (*Sauroleishmania*) sp.).

Through preliminary determination of identity using BLASTn, the total prevalence of *Leishmania* DNA detected via PCR was 46.7% (7/15). 5 (33.3%) snakes were detected as positive for *cyt b*, 4 (26.7%) snakes for *Hsp70* and 2 (13.3%) for *ITS1*. Considering the sensitivity of different loci, two samples (Guo4707 and Guo4708) from P1 and P2 were amplified successfully in all three loci, while other samples were detected by only one locus in this study. As shown in Fig 1, the snakes from P1, 2, 3, 4, 5, 6 and 9 with colored spots were *Leishmania*-positive.

### Sequence characteristics

After the haplotypes were defined, 10 *cyt b* sequences, 7 *Hsp70* sequences and 10 *ITS1* sequences were obtained in this study and deposited in the GenBank database under Accession No. MK330198-MK330207, MK330208-MK330214 and MK300708-MK300717, respectively (Table 2). PCR amplification of each target locus resulted in amplicons of the expected sizes as follows: *cyt b* (543 bp), *Hsp70* (738–767 bp), and *ITS1* (328–334 bp). The GC contents were 23.4% for *cyt b*, 64.9% for *Hsp70* and 41.8% for *ITS1*, respectively. Thus, both their lengths and their GC contents are within the range of corresponding sequences of other *Leishmania* species.

**Table 2:** List of the snake samples, origin, isolated *Leishmania* spp. and GenBank accession numbers.

Snake species	Voucher number	Origin	Isolated *Leishmania* spp.	Haplotype number	GenBank accession	Sequence length (bp)
**(A) *cyt b***						
*Psammophis lineolatus*	Guo4707	P1	*L*. *donovani*	C1	MK330198	543
			*L*. (*Sauroleishmania*) sp.	C6	MK330199	543
*Gloydius halys*	Guo4708	P2	*L*. *donovani*	C1	MK330200	543
*Natrix tessellata*	Guo4709	P3	*L*. *donovani*	C2	MK330201	543
			*L*. *turanica*	C4	MK330202	543
			*L*. (*Sauroleishmania*) sp.	C5	MK330203	543
			*L*. (*Sauroleishmania*) sp.	C6	MK330204	543
*Psammophis lineolatus*	Guo4710	P4	*L*. *donovani*	C3	MK330205	543
			*L*. *turanica*	C4	MK330206	543
*Psammophis lineolatus*	Guo6995	P9	*L*. (*Sauroleishmania*) sp.	C6	MK330207	543
**(B) *Hsp70***						
*Psammophis lineolatus*	Guo4707	P1	*L*. *donovani*	H1	MK330208	738
			*L*. *infantum*	H2	MK330209	738
*Gloydius halys*	Guo4708	P2	*L*. *infantum*	H2	MK330210	738
*Psammophis lineolatus*	Guo4023	P6	*L*. (*Sauroleishmania*) sp.	H3	MK330211	767
			*L*. (*Sauroleishmania*) sp.	H4	MK330212	766
			*L*. (*Sauroleishmania*) sp.	H5	MK330213	767
*Psammophis lineolatus*	Guo4026	P5	*L*. (*Sauroleishmania*) sp.	H5	MK330214	767
**(C) *ITS1***						
*Psammophis lineolatus*	Guo4707	P1	*L*. (*Sauroleishmania*) sp.	I1	MK300708	334
			*L*. (*Sauroleishmania*) sp.	I2	MK300709	328
			*L*. (*Sauroleishmania*) sp.	I3	MK300710	332
			*L*. (*Sauroleishmania*) sp.	I4	MK300711	330
			*L*. (*Sauroleishmania*) sp.	I5	MK300712	330
			*L*. (*Sauroleishmania*) sp.	I6	MK300713	330
			*L*. (*Sauroleishmania*) sp.	I7	MK300714	330
*Gloydius halys*	Guo4708	P2	*L*. (*Sauroleishmania*) sp.	I8	MK300715	330
			*L*. (*Sauroleishmania*) sp.	I9	MK300716	332
			*L*. (*Sauroleishmania*) sp.	I10	MK300717	328

### Phylogenetic relationship

Prior to the phylogenetic analyses, the most adequate models of nucleotide substitution were selected by PartitionFinder v2.1.1: GTR +G and GTR+I+G for *cyt b*, HKY+I and GTR for *Hsp70*, K80+G for *ITS1*. Using Bayesian inference method, the trees showed similar phylogenetic topology for all three loci supported by posterior probability (PP) values.

**Fig 2:**
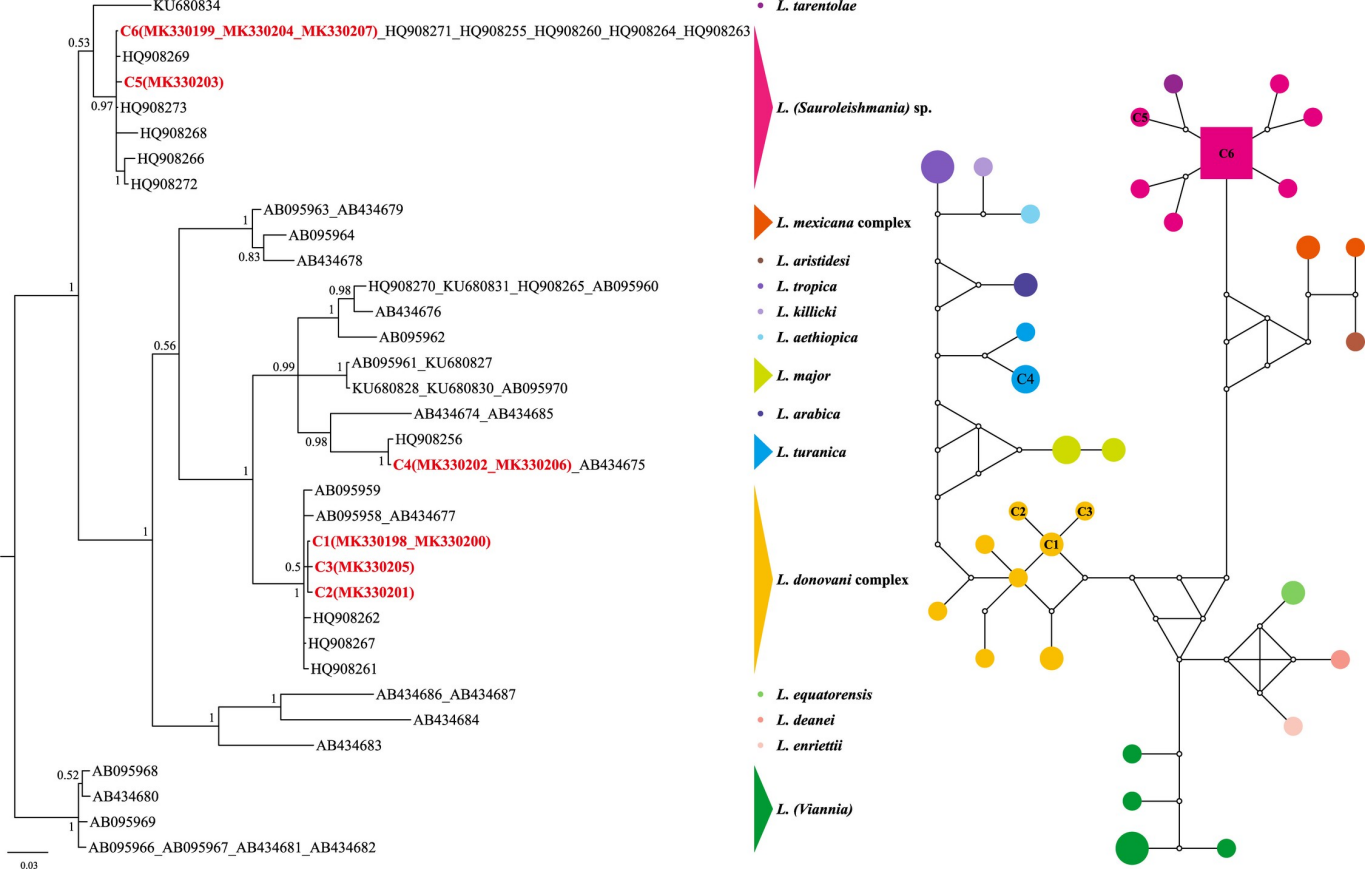
The majority-rule consensus tree (midpoint rooted) of *Cyt b* inferred from Bayesian inference by using MrBayes v.3.2, and the corresponding median-joining network implemented by NETWORK v5.0.0.3. Different colors represent the corresponding *Leishmania* species obtained in this study; numbers at the nodes of the trees are the posterior probability (PP) values; circles of the networks indicate the haplotypes and squares for the central haplotypes, the small hollow circles indicate median vectors; the information of reference sequences see S1A Table.

Regarding the BI tree inferred from each locus, the phylogenetic analyses inferred from the aligned matrix of *cyt b* (Fig 2) separated two distance clades, the *Viannia* isolates apparently formed an independent clade (PP = 1.0) outside the clusters of those other species (PP = 1.0), while the subgenera *Leishmania* and *Sauroleishmania* were still separated into different branches (PP = 1.0 and 0.53, respectively). The isolates C1, C2 and C3 clustered with *L*. *donovani* and *L*. *infantum* (PP = 1.0). The isolate C4 shared an identical *cyt b* sequence to a *L*. *turanica* isolate and they were grouped with *L*. *arabica*, *L*. *major*, *L*. *tropica*, *L*. *aethiopica* and *L*. *killicki* in a subclade (PP = 0.99) separated with *L*. *donovani* complex. The isolate C6 shared identical sequences with 5 Chinese *Leishmania* sp. isolates from Yang et al. [29] clustered in a separated clade (PP = 0.96) together with C5 and other *L*. (*Sauroleishmania*) sp.

**Fig 3:**
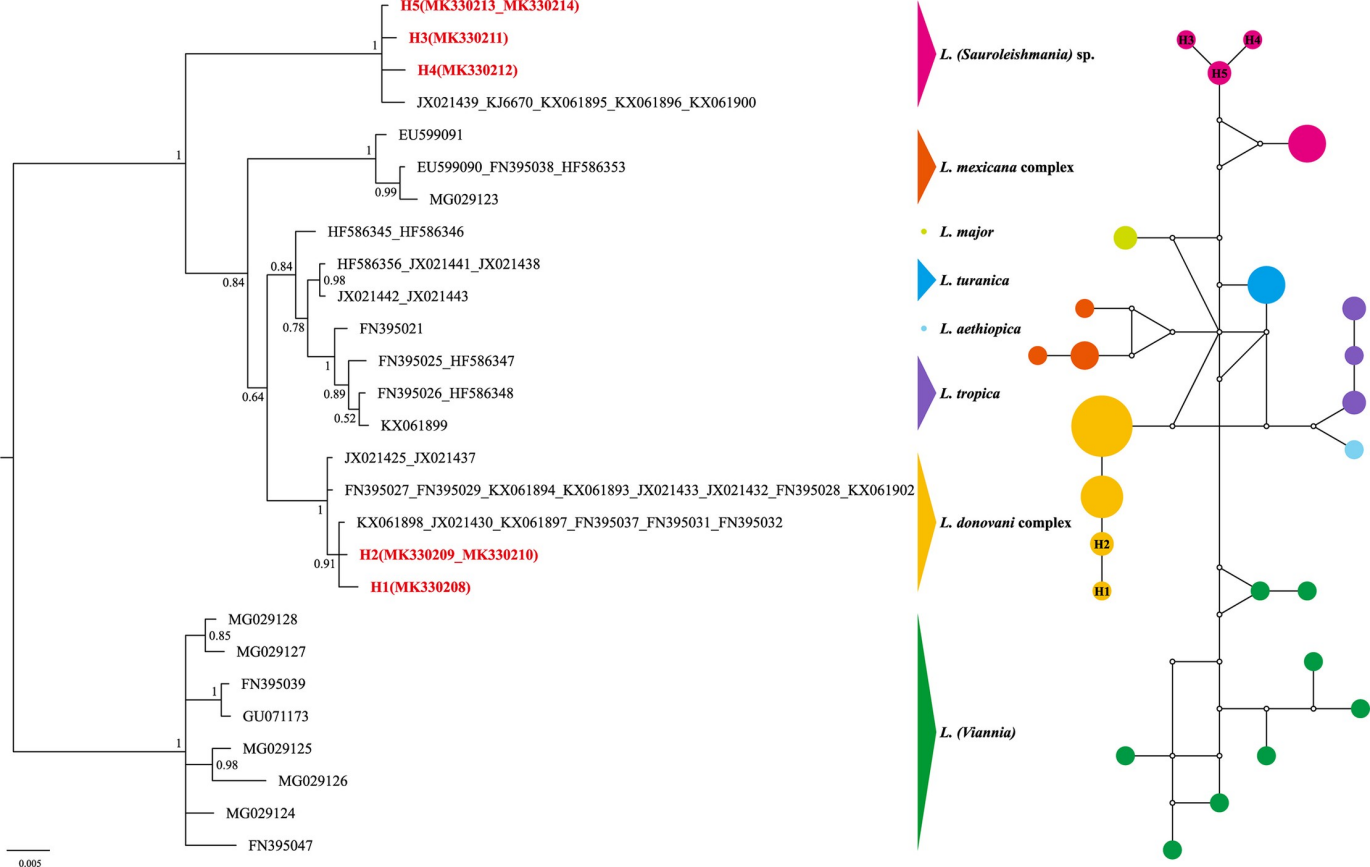
The majority-rule consensus tree (midpoint rooted) and the median-joining network based on *Hsp70*. Different colors represent the corresponding *Leishmania* species obtained in this study; numbers at the nodes of the trees are the posterior probability (PP) values; circles of the networks indicate the haplotypes and squares for the central haplotypes, the small hollow circles indicate median vectors; the information of reference sequences see S1B Table.

The *Hsp70* analysis (Fig 3) showed a similar structure to the phylogenetic tree of *cyt b*: *Viannia* (PP = 1.0), *Sauroleishmania* (PP = 1.0) and *Leishmania* (PP = 0.84). The isolates H1 and H2 searching by BLASTn (Megablast) revealed over 98% identity with *L*. *donovani* and *L*. *infantum* respectively, both of them were nested within *L*. *donovani* complex. In this subclade, the isolates of other species of *Leishmania* subgenus (except *L*. *mexicana* complex clustered by the PP 1.0) were not in reciprocally monophyletic groups. The isolates H3, H4 and H5 clustered in a separated clade (PP = 1.0) with *L*. (*Sauroleishmania*) sp. from China.

**Fig 4:**
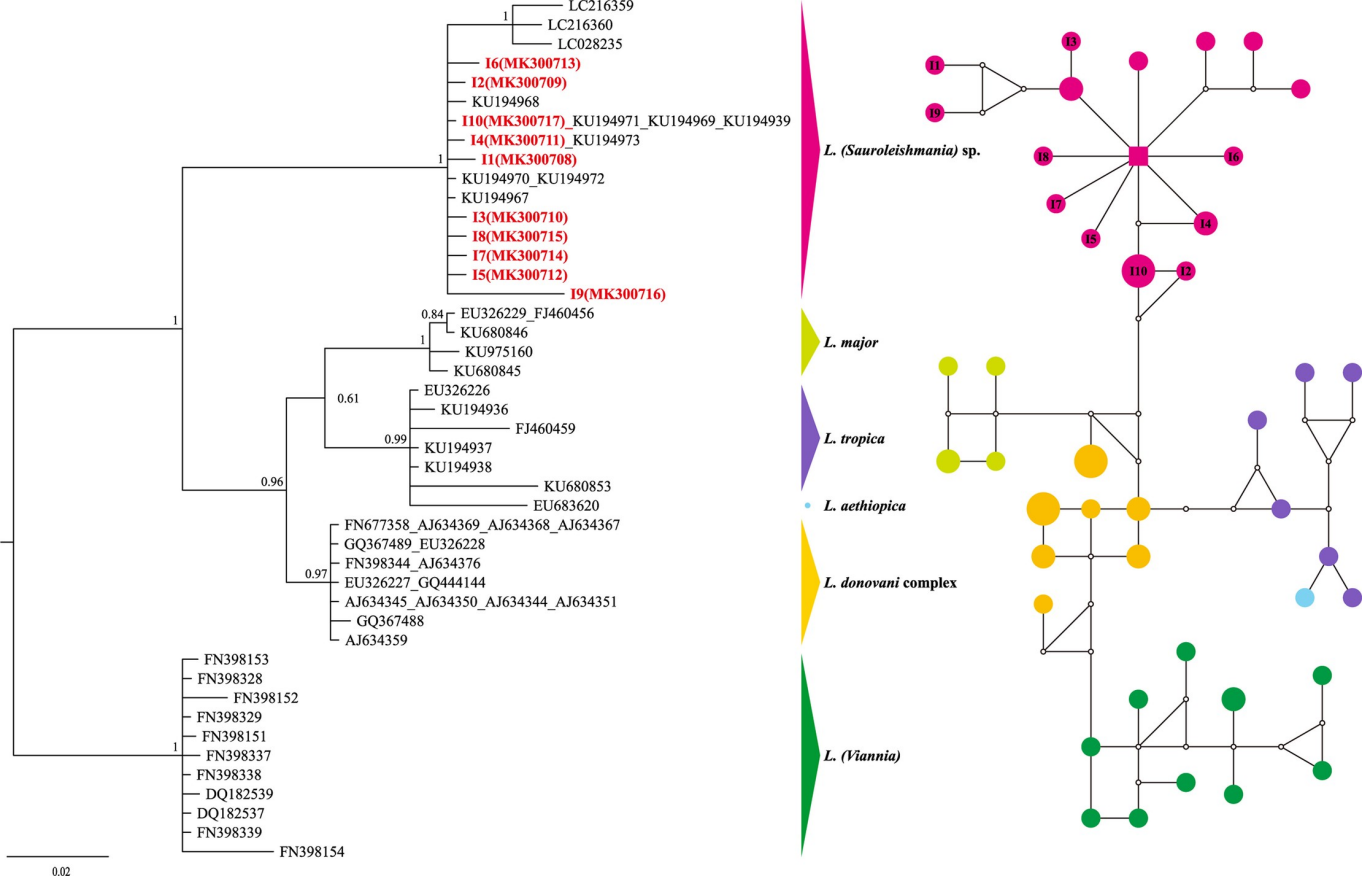
The majority-rule consensus tree (midpoint rooted) and the median-joining network based on *ITS1*. Different colors represent the corresponding *Leishmania* species obtained in this study; numbers at the nodes of the trees are the posterior probability (PP) values; circles of the networks indicate the haplotypes and squares for the central haplotypes, the small hollow circles indicate median vectors; the information of reference sequences see S1C Table.

For the *ITS1*-5.8S region, the phylogenetic tree as Fig 4 shown was constructed by three clades with high support (PP = 1.0, 0.96 and 1.0, respectively), corresponding to the three subgenera *Viannia*, *Leishmania*, and *Sauroleishmania*. All the 10 fragments from snakes formed a strongly supported cluster with *L*. (*Sauroleishmania*) sp. isolated from lizards [21] (PP = 1.0), and revealed a close relationship to isolates from *Sergentomyia minuta* in Europe.

### Median joining network

To get additional insight into the relationships among the strains, we analyzed our data sets using the Median Joining algorithm network approach. The MJ analysis of three regions was congruent with the topology described in the BI trees, and no haplotype was shared among the species groups.

The network of *cyt b* in Fig 2 showed that the isolate C6 seemed to be the central haplotype. C1 (MK330198 isolated from two snakes captured at P1 and MK330200 from P2), together with C2 (MK330201 from P3) and C3 (MK330205 from P4) were most closely related to the central HQ908267, within 2 mutational steps. C4 (from P3 and P4) shared the same haplotype with one strain of *L*. *turanica* from Russia, and only 2 and 3 steps away from the Chinese strains *L*. *turanica* (from Xinjiang). The *L*. (*Sauroleishmania*) sp. isolate C6 (from P1 and P9) as the central haplotype shared the same sequences to *L*. (*Sauroleishmania*) sp. from China with a wide geographical distribution, including Shandong (HQ908255), Sichuan (HQ908260, HQ908264), Gansu (HQ908263, HQ908271).

For the *Hsp70* region, the haplotype network of Fig 3 could intuitively reflect the genetically small distances between the obtained sequences H1 (MK330208 isolated from P1), H2 (MK330209 and MK330210 from P1 and P2, respectively) and the reference *L*. *donovani* and *L*. *infantum* strains from India, Sudan and the northwest of China. The haplotype H5 shared by the isolates from P5 (MK330213) and P6 (MK330214) was the interior haplotype of *L*. (*Sauroleishmania*) sp. and may be older than other top haplotypes H3 (MK330211) or H4 (MK330212) which were both from P6). It reflected a close distance between JX021439 (Xinjiang, China) and the interior haplotype as three mutational steps.

As shown in the haplotype network of *ITS1*-5.8S in Fig 4, the *L*. (*Sauroleishmania*) sp. group was centered around haplotype KU194967 (Dunhuang, China). All haplotypes found in this group of *Sauroleishmania* isolated from lizards of the northwest of China were very closely related to each other. One strain from Tuokexun (KU194973) shared the same haplotype with I4 (MK300711 from P1). Similarly, two strains from Dunhuang (KU194969, KU194971) and another one from Lukchun (KU194939) shared the same haplotype with I10 (MK300709 from P2). In addition, three strains isolated from *Sergentomyia minuta* of Europe shared a close distance with the *Sauroleishmania* group as less than 3 steps.

## Discussion

This study was the first time to detect the infection of *Leishmania* in snakes by DNA sequencing. Our result showed that the snakes from 7 loci of the 10 study areas were detected positive for *Leishmania*. Yopurga county, Hami and Dunhuang are the DT-ZVL epidemic foci [5], while for Shawan and Texes county current data are lacking.

Three *Leishmania* species (including *L*. *donovani*, *L*. *turanica* and *L*. (*Sauroleishmania*) sp.) were detected from one snake (Guo4709, *Natrix tessellate*) which was captured from Texes, whereas only 1–2 species were detected in the other *Leishmania*-positive snakes. *L*. (*Sauroleishmania*) sp. was the most widely distributed species. Owing to the limit of the sample size, the other two *Leishmania* species (*L*. *donovani* and *L*. *turanica*) were lack of significant geographic specificity in this study.

The effect of many factors (e.g., the condition of specimen, the DNA extraction methodology, the length of amplified gene sequences and the PCR protocols) would cause different rates in PCR positivity. Amplification of multiple gene sequences could significantly increase the comprehensiveness of the *Leishmania* infections to be investigated in snakes. Various genetic loci had been developed to detect *Leishmania*, such as kinetoplast DNA (kDNA) genes, nuclear DNA (nDNA) genes and ribosomal RNA (rRNA) genes [28,49,50]. *Cyt b*, one of the cytochromes encoded on the kinetoplast DNA maxicircle, is considered one of the most useful markers that had delimitated most tested *Leishmania* species for phylogenetic work. *Hsp70* gene, an antigen gene on the chromosome, is the most optimal marker for the species discrimination and phylogenetic inference. *ITS1*, one of the highly variable regions of rRNA gene, have been successfully used to resolve taxonomical questions and to determine phylogenetic affinities among closely related *Leishmania* species. Therefore, three genetic loci were amplified in this study and the positivity rates were different indeed. For two samples from Shawan and Tekes, *L*. (*Sauroleishmania*) sp. and *L*. *donovani* were identified by all the three genetic loci while for other samples only one of the three genetic loci could be amplified. It was suggested that different *Leishmania* primers might have different selection preferences for the gene amplification. At the same time, the use of different primers to amplify the corresponding gene fragments did significantly improve the detection rate of infected snake samples.

The spreading mechanism of *Leishmania* in reptiles is still debated. Wilson & Southgate assumed that transmission was achieved by the reptile host eating an infected sand fly [51,52]. Nevertheless, there are species of *phlebotomine* that are known to feed on reptiles [53,54]. The same as lizards, it was reasonable that snakes could be infected naturally. In view of the small sample size per location and species, the calculation of infection rates does not make much sense. *Leishmania* DNA was detected in all three species of snakes captured in endemic foci of VL, even though the sample size of both *Gloydius halys* and *Natrix tessellate* was 1. Therefore, a bigger sample size would be necessary for further studies to enrich our understanding on natural infections of *Leishmania* parasites in snakes.

One of the most noteworthy findings in the present study was that two mammalian *Leishmania* taxa, i.e., *L*. *donovani* complex and *L*. *turanica* appeared to occur also in snakes. Previously, Zhang et al. had a similar discovery in lizards [21]. They believe that lizards may be putative reservoirs for human leishmaniasis because of the common ancestry of the haplotypes obtained for *L*. *tropica* and clinical samples. At present, apart from the above study, there are few studies of mammalian parasites natural infections in reptiles. Belova [12] carried out intensive research on reptilian and mammalian *Leishmania* and found that the mammalian parasites (such as *L*. *donovani*, *L*. *tropica*, and some other promastigotes recovered from sandflies) were infective to lizards. To determine whether reptiles might be reservoir hosts for human leishmaniasis, it is necessary to sample of *Leishmania* strains from VL patients and from reptiles in the epidemic foci and to compare them by phylogenetic analyses.

On the other hand, the *L*. (*Sauroleishmania*) sp. isolated from snakes in this study shared the same haplotypes or were located in close proximity to strains from VL patients or canines in China as shown by the networks. Indeed, the Chinese *L*. (*Sauroleishmania*) sp. isolates were speculated to be closely related to the lizard-infected strains, *L*. *tarentolae*, on the basis of COII [55] and *cyt b* [31] genes. Analogously, *L*. *adleri* was found to cause transient CL in humans by Manson-Bahr & Heisch [56], and could infect rodents from which the strains were isolated by Adler [23] and Coughlan et al. [57]. *L*. *tarentolae* was chosen as a candidate vaccine against VL in virtue of the character that the parasites could invade in human macrophages and exist as amastigotes in mammals with slower replication [24]. These studies seem to be contradictory with the former which indicated that *Sauroleishmania* was not pathogenic to human [12]. Since the Chinese strains from patients or canines were isolated in the 70s to 90s of the last century, medical records were no longer available, and we could not confirm whether the patients got leishmaniasis due to *Sauroleishmania* or co-infection with other mammalian *Leishmania*. Thus, to further explore the pathogenicity of *Sauroleishmania*, it is recommended to isolate and culture the promastigotes from liver or blood of reptiles and study the virulence to mammals.

In conclusion, the present study is the first which investigates natural *Leishmania* spp. infections of snakes in the northwest of China. The species identification was primarily achieved by comparisons of the obtained sequences with the GenBank database and determined the belongs with phylogenetic and network analyses, including *L*. (*Sauroleishmania*) sp., *L*. *donovani*, *L*. *turanica*. For the first time mammalian *Leishmania* were discovered in snakes, after the similar finding in lizards. The *Sauroleishmania* strains in this study were closely related to clinical isolates, which suggests us to be cautious about the widely accepted view that *Sauroleishmania* is nonpathogenic to human. These findings invite us to ponder the importance of snakes in the disease cycle. However, more snake samples in epidemic foci and the pathogenic features of *Sauroleishmania* promastigotes are required for indicating their epidemiological involvement.

## Supporting information

S1 Table Accession numbers for *Leishmania* sequences downloaded from GenBank and used for the phylogenetic analyses presented in Fig 2. (DOCX)
