# Correction: Multi-locus characterization and phylogenetic inference of *Leishmania* spp. in snakes from Northwest China

**DOI:** 10.1371/journal.pone.0221307

**Published:** 2019-08-13

**Authors:** The PLOS ONE Staff

The Author Contributions for Minli Chen are incorrect. The correct contributions for Minli Chen are: Investigation, Software. The publisher apologizes for the error.
