# Supplementary material for: Multi-locus characterization and phylogenetic inference of Leishmania spp. in snakes from Northwest China
Source: PLoS One. 2019 Apr 25;14(4):e0210681. doi: 10.1371/journal.pone.0210681 (PMC6483563; doi:10.1371/journal.pone.0210681)
Supplement: S1 Table — (DOCX) [file pone.0210681.s001.docx]

**S1A Table. Accession numbers for *Leishmania* *cyt b* sequences downloaded from GenBank and used for the phylogenetic analyses presented in Fig 2**

| GenBank accession number | Species | Isolate (WHO code) | Origin | Host | Reference |
| --- | --- | --- | --- | --- | --- |
| AB095958 | *L. infantum* | MHOM/TN/80/IPT1 | Tunisia | *Homo sapiens* | Luyo-Acero,G.E. (2004) |
| AB095959 | *L. chagasi* | MHOM/BR/74/PP75 | Brazil | *Homo sapiens* | Luyo-Acero,G.E. (2004) |
| AB095960 | *L. tropica* | MHOM/SU/58/Strain OD | Russia | *Homo sapiens* | Luyo-Acero,G.E. (2004) |
| AB095961 | *L. major* | MHOM/SU/73/5ASKH | Russia | *Homo sapiens* | Luyo-Acero,G.E. (2004) |
| AB095962 | *L. aethiopica* | MHOM/ET/72/L100 | Ethiopia | *Homo sapiens* | Luyo-Acero,G.E. (2004) |
| AB095963 | *L. mexicana* | MHYC/BZ/62/M379 | Brazil | *Homo sapiens* | Luyo-Acero,G.E. (2004) |
| AB095964 | *L. amazonensis* | MHOM/BR/73/M2269 | Brazil | *Homo sapiens* | Luyo-Acero,G.E. (2004) |
| AB095966 | *L. braziliensis* | MHOM/BR/75/M2904 | Brazil | *Homo sapiens* | Luyo-Acero,G.E. (2004) |
| AB095967 | *L. braziliensis* | MHOM/EC/88/INH-03 | Ecuador | *Homo sapiens* | Luyo-Acero,G.E. (2004) |
| AB095968 | *L. panamensis* | MHOM/BR/71/LS94 | Brazil | *Homo sapiens* | Luyo-Acero,G.E. (2004) |
| AB095969 | *L. guyanensis* | MHOM/BR/75/M4147 | Brazil | *Homo sapiens* | Luyo-Acero,G.E. (2004) |
| AB095970 | *L. cf. major* | MHOM/EC/88/PT-115 | Ecuador | *Homo sapiens* | Luyo-Acero,G.E. (2004) |
| AB434674 | *L. arabica* | MPSA/SA/83/JISH220 | Saudi Arabia | *Homo sapiens* | Asato,Y. (2009) |
| AB434675 | *L. turanica* | MRHO/SU/80/CLONE3720 | Russia | *Homo sapiens* | Asato,Y. (2009) |
| AB434676 | *L. killicki* | MHOM/TN/86/LEM163 | Tunisia | *Homo sapiens* | Asato,Y. (2009) |
| AB434677 | *L. donovani archibaldi* | MHOM/ET/72/GEBRE1 | Ethiopia | *Homo sapiens* | Asato,Y. (2009) |
| AB434678 | *L. aristidesi* | MORY/PA/69/GML | Panama | *Homo sapiens* | Asato,Y. (2009) |
| AB434679 | *L. pifanoi* | MHOM/VE/57/LL1 | Venezuela | *Homo sapiens* | Asato,Y. (2009) |
| AB434680 | *L. shawi* | MHOM/BR/79/M15065 | Brazil | *Homo sapiens* | Asato,Y. (2009) |
| AB434681 | *L. braziliensis* | MHOM/BR/00/LTB300 | Brazil | *Homo sapiens* | Asato,Y. (2009) |
| AB434682 | *L. braziliensis* | MHOM/BR/75/M2903 | Brazil | *Homo sapiens* | Asato,Y. (2009) |
| AB434683 | *L. enriettii* | MCAV/BR/45/L88 | Brazil | *Homo sapiens* | Asato,Y. (2009) |
| AB434684 | *L. deanei* | MCOE/BR/74/M2674 | Brazil | *Homo sapiens* | Asato,Y. (2009) |
| AB434685 | *L. arabica* | MPSA/SA/83/JISH220 | Saudi Arabia | *Homo sapiens* | Asato,Y. (2009) |
| AB434686 | *L. equatorensis* | MCOH/EC/82/LSP-1 | Ecuador | *Homo sapiens* | Asato,Y. (2009) |
| AB434687 | *L. equatorensis* | MSCI/EC/82/LSP-2 | Ecuador | *Homo sapiens* | Asato,Y. (2009) |
| HQ908255 | *L.* (*Sauroleishmania*) sp. | MHOM/CN/84/SD1 | China: Shandong province | *Homo sapiens* | Yang,B.B. (2013) |
| HQ908256 | *L. turanica* | MRHO/CN/88/KXG-2 | China: Xinjiang Uygur Autonomous Region | *Rhombomys opimus* | Yang,B.B. (2013) |
| HQ908260 | *L.* (*Sauroleishmania*) sp. | MHOM/CN/90/SC10H2 | China: Sichuan province | *Homo sapiens* | Yang,B.B. (2013) |
| HQ908261 | *L. infantum* | MHOM/CN/93/GS7 | China: Gansu province | *Homo sapiens* | Yang,B.B. (2013) |
| HQ908262 | *L. donovani* | MCAN/CN/60/GS1 | China: Gansu province | Canine | Yang,B.B. (2013) |
| HQ908263 | *L.* (*Sauroleishmania*) sp. | MHOM/CN/89/GS6 | China: Gansu province | - | Yang,B.B. (2013) |
| HQ908264 | *L.* (*Sauroleishmania*) sp. | MHOM/CN/86/SC6 | China: Sichuan province | *Homo sapiens* | Yang,B.B. (2013) |
| HQ908265 | *L.* (*Sauroleishmania*) sp. | MHOM/CN/84/JS1 | China: Jiangsu province | *Homo sapiens* | Yang,B.B. (2013) |
| HQ908266 | *L.* (*Sauroleishmania*) sp. | MHOM/CN/80/XJ801 | China: Xinjiang Uygur Autonomous Region | *Homo sapiens* | Yang,B.B. (2013) |
| HQ908267 | *L. donovani* | IPHL/CN/77/XJ771 | China: Xinjiang Uygur Autonomous Region | Sand fly | Yang,B.B. (2013) |
| HQ908268 | *L.* (*Sauroleishmania*) sp. | MCAN/CN/86/SC9 | China: Sichuan province | *Homo sapiens* | Yang,B.B. (2013) |
| HQ908269 | *L.* (*Sauroleishmania*) sp. | MHOM/CN/89/GS5 | China: Gansu province | *Homo sapiens* | Yang,B.B. (2013) |
| HQ908270 | *L. tropica* | MHOM/SU/74/K27 | USSR | *Homo sapiens* | Yang,B.B. (2013) |
| HQ908271 | *L.* (*Sauroleishmania*) sp. | MHOM/CN/84/GS3 | China: Gansu province | *Homo sapiens* | Yang,B.B. (2013) |
| HQ908272 | *L.* (*Sauroleishmania*) sp. | MHOM/CN/90/SC11 | China: Sichuan province | *Homo sapiens* | Yang,B.B. (2013) |
| HQ908273 | *L.* (*Sauroleishmania*) sp. | MHOM/CN/83/GS2 | China: Gansu province | *Homo sapiens* | Yang,B.B. (2013) |
| KU680827 | *L. major* | MHOM/SU/73/5ASKH | Russia | *Homo sapiens* | Fotouhi-Ardakani,R. (2016) |
| KU680828 | *L. major* | MRHO/IR/75/ER | Iran | *Homo sapiens* | Fotouhi-Ardakani,R. (2016) |
| KU680830 | *L. major* | MHOM/IL/80/friedlin | Italy | *Homo sapiens* | Fotouhi-Ardakani,R. (2016) |
| KU680831 | *L. tropica* | MHOM/SU/74/K27 | Russia | *Homo sapiens* | Fotouhi-Ardakani,R. (2016) |
| KU680834 | *L. tarentolae* | Strain p10 | Germany | - | Fotouhi-Ardakani,R. (2016) |

**S1B Table. Accession numbers for *Leishmania* *Hsp70* sequences downloaded from GenBank and used for the phylogenetic analyses presented in Fig 3**

| GenBank accession number | Species | Isolate (WHO code) | Origin | Host | Reference |
| --- | --- | --- | --- | --- | --- |
| EU599090 | *L. amazonensis* | MHOM/BR/73/M2269 | Brazil | *Homo sapiens* | Fraga,J. (2010) |
| EU599091 | *L. mexicana* | MNYC/BZ/62/M379 | Brazil | *Homo sapiens* | Fraga,J. (2010) |
| FN395021 | *L. aethiopica* | MHOM/ET/72/L100 | Ethiopia | *Homo sapiens* | Fraga,J. (2010) |
| FN395025 | *L. tropica* | MHOM/IN/79/DD7 | India | - | Fraga,J. (2010) |
| FN395026 | *L. tropica* | MHOM/KE/81/NLB_030B | Kenya | - | Fraga,J. (2010) |
| FN395027 | *L. donovani* | MHOM/SD/68/1S | Sudan | - | Fraga,J. (2010) |
| FN395028 | *L. donovani* | MHOM/IN/00/DEVI | India | *Homo sapiens* | Fraga,J. (2010) |
| FN395029 | *L. donovani* | MHOM/SD/82/Gilani | Sudan | *Homo sapiens* | Fraga,J. (2010) |
| FN395031 | *L. infantum* | MHOM/MT/85/Buck | Malta | *Homo sapiens* | Fraga,J. (2010) |
| FN395032 | *L. infantum* | MHOM/PT/00/IMT260 | Portugal | *Homo sapiens* | Fraga,J. (2010) |
| FN395037 | *L. chagasi* | MHOM/BR/07/ARL | Brazil | - | Fraga,J. (2010) |
| FN395038 | *L. mexicana* | MHOM/PE/02/LH2312 | Peru | - | Fraga,J. (2010) |
| FN395039 | *L. braziliensis* | MHOM/BO/--/CUM180 | Bolivia | - | Fraga,J. (2010) |
| FN395047 | *L. lainsoni* | MHOM/BO/95/CUM71 | Bolivia | - | Fraga,J. (2010) |
| GU071173 | *L. braziliensis* | MHOM/BR/1975/M2903 | Brazil | *Homo sapiens* | da Silva,L.A. (2010) |
| GU071188 | *L. utingensis* | ITUB/BR/1977/M4964 | Brazil | - | da Silva,L.A. (2010) |
| HF586345 | *L. major* | MHOM/BF/2004/REN04-8 | Burkina Faso | - | Van der Auwera,G. (2013) |
| HF586346 | *L. major* | MHOM/SD/2003/LCB33 | Sudan | - | Van der Auwera,G. (2013) |
| HF586347 | *L. tropica* | MHOM/MA/88/LEM1314 | Morocco | *Homo sapiens* | Van der Auwera,G. (2013) |
| HF586348 | *L. tropica* | MHOM/YE/86/LEM1015 | Yemen | *Homo sapiens* | Van der Auwera,G. (2013) |
| HF586353 | *L. amazonensis* | MHOM/CO/82/CELIS | Colombia | *Homo sapiens* | Van der Auwera,G. (2013) |
| HF586356 | *L. turanica* | MRHO/SU/95/T-9551R | Russia | Rodent | Van der Auwera,G. (2013) |
| JX021425 | *L. donovani* | IWUI/CN/77/771 | China: Xinjiang | Sand fly | Zhang,C.Y. (2013) |
| JX021430 | *L. donovani* | MHOM/CN/86/SC9 | China: Sichuan | Canine | Zhang,C.Y. (2013) |
| JX021432 | *L. infantum* | MHOM/CN/94/KXG-LIU | China: Xinjiang | *Homo sapiens* | Zhang,C.Y. (2013) |
| JX021433 | *L. infantum* | MHOM/CN/93/KXG-XU | China: Xinjiang | *Homo sapiens* | Zhang,C.Y. (2013) |
| JX021437 | *L. donovani* | IMJW/CN/87/KXG-65 | China: Xinjiang | Sand fly | Zhang,C.Y. (2013) |
| JX021438 | *L. turanica* | IAND/CN/90/KXG-R | China: Xinjiang | Sand fly | Zhang,C.Y. (2013) |
| JX021439 | *L.* (*Sauroleishmania*) sp. | IARP/CN/90/KXG-E | China: Xinjiang | Sand fly | Zhang,C.Y. (2013) |
| JX021441 | *L. turanica* | MRHO/CN/90/KXG-57 | China: Xinjiang | rodent | Zhang,C.Y. (2013) |
| JX021442 | *L. turanica* | MRHO/CN/92/QITAI-15 | China: Xinjiang | rodent | Zhang,C.Y. (2013) |
| JX021443 | *L. turanica* | MRHO/CN/87/KXG-11 | China: Xinjiang | rodent | Zhang,C.Y. (2013) |
| KJ667088 | *L.* (*Sauroleishmania*) sp. | MHOM/CN/85/GS4 | China: Gansu province | - | Guan,W. (Unpublished) |
| KJ667089 | *L.* (*Sauroleishmania*) sp. | MHOM/GS/54/3# | China: Gansu province | - | Guan,W. (Unpublished) |
| KJ667090 | *L.* (*Sauroleishmania*) sp. | MHOM/CN/89/GS5 | China: Gansu province | *Homo sapiens* | Guan,W. (Unpublished) |
| KJ667091 | *L.* (*Sauroleishmania*) sp. | MHOM/GS/90/SC10H2 | China: Sichuan province | *Homo sapiens* | Guan,W. (Unpublished) |
| KJ667092 | *L.* (*Sauroleishmania*) sp. | MHOM/GS/89/GS6 | China: Gansu province | - | Guan,W. (Unpublished) |
| KJ667093 | *L.* (*Sauroleishmania*) sp. | MHOM/CN/83/GS2 | China: Gansu province | *Homo sapiens* | Guan,W. (Unpublished) |
| KJ667094 | *L.* (*Sauroleishmania*) sp. | MCAN/CN/86/SC9 | China: Sichuan province | *Homo sapiens* | Guan,W. (Unpublished) |
| KJ667095 | *L.* (*Sauroleishmania*) sp. | MHOM/GS/84/SD1 | China: Shandong province | *Homo sapiens* | Guan,W. (Unpublished) |
| KX061893 | *L. donovani* | IMJW/CN/91/KXG-918 | China: Xinjiang Uygur Autonomous Region | - | Yuan,D.M. (2016) |
| KX061894 | *L. donovani* | MHOM/IN/80/DD8 | India | *Homo sapiens* | Yuan,D.M. (2016) |
| KX061895 | *L.* (*Sauroleishmania*) sp. | GL | China: Gansu province | *Homo sapiens* | Yuan,D.M. (2016) |
| KX061896 | *L.* (*Sauroleishmania*) sp. | MHOM/CN/90/SC10H2 | China: Sichuan province | *Homo sapiens* | Yuan,D.M. (2016) |
| KX061897 | *L. infantum* | MCAN/CN/08/Cy | China | - | Yuan,D.M. (2016) |
| KX061898 | *L. infantum* | MCAN/CN/90/WenChuan | China: Sichuan province | - | Yuan,D.M. (2016) |
| KX061899 | *L. tropica* | MHOM/SU/74/K27 | Russia | *Homo sapiens* | Yuan,D.M. (2016) |
| KX061900 | *L.* (*Sauroleishmania*) sp. | SD | China: Shandong province | - | Yuan,D.M. (2016) |
| KX061902 | *L. donovani* | IMJW/CN/92/KXG-927 | China: Xinjiang Uygur Autonomous Region | - | Yuan,D.M. (2016) |
| MG029123 | *L. amazonensis* | MHOM/BR/73/M2269 | Brazil | - | Espada,C.R. (2018) |
| MG029124 | *L. lindenbergi* | MHOM/BR/96/M15732 | Brazil | - | Espada,C.R. (2018) |
| MG029125 | *L. utingensis* | ITUB/BR/77/M4964 | Brazil | - | Espada,C.R. (2018) |
| MG029126 | *L. naiffi* | IAYR/B5/86/EO337 | Brazil | - | Espada,C.R. (2018) |
| MG029127 | *L. shawi* | MCEB/BR/84/M8408 | Brazil | - | Espada,C.R. (2018) |
| MG029128 | *L. guyanensis* | MHOM/BR/2002/ACVJ | Brazil | - | Espada,C.R. (2018) |

**S1C Table. Accession numbers for *Leishmania* *ITS1* sequences downloaded from GenBank and used for the phylogenetic analyses presented in Fig 4**

| GenBank accession number | Species | Isolate (WHO code) | Origin | Host | Reference |
| --- | --- | --- | --- | --- | --- |
| AJ634344 | *L. infantum* | MHOM/PT/00/IMT260 | Portugal | *Homo sapiens* | Kato,H. (2011) |
| AJ634345 | *L. infantum* | MHOM/CN/54/Peking | China | *Homo sapiens* | Kato,H. (2011) |
| AJ634350 | *L. infantum* | MHOM/MT/85/BUCK | Malta | *Homo sapiens* | Kato,H. (2011) |
| AJ634351 | *L. infantum* | MHOM/FR/80/LEM189 | France | *Homo sapiens* | Kato,H. (2011) |
| AJ634359 | *L. donovani* | MHOM/SD/97/LEM3463 | Sudan | *Homo sapiens* | Kato,H. (2011) |
| AJ634367 | *L. donovani* | MHOM/ET/72/GEBRE1 | Ethiopia | *Homo sapiens* | Kato,H. (2011) |
| AJ634368 | *L. donovani* | MHOM/SD/93/338 | Sudan | *Homo sapiens* | Kato,H. (2011) |
| AJ634369 | *L. infantum* | MHOM/SD/82/GILANI | Sudan | *Homo sapiens* | Kato,H. (2011) |
| AJ634376 | *L. donovani* | MHOM/IN/00/DEVI | India | *Homo sapiens* | Kato,H. (2011) |
| DQ182537 | *L. braziliensis* | MHOM/BR/84/LTB300 | Brazil | - | Rotureau,B. (2006) |
| DQ182539 | *L. guyanensis* | MHOM/GF/2003/LBC40 | French Guiana | - | Rotureau,B. (2006) |
| EU326226 | *L. tropica* | MHOM/SU/60/OD | Russia | *Homo sapiens* | Thakur,S. (Direct Submission, 2007) |
| EU326227 | *L. infantum* | MHOM/BR/74/PP75 | Brazil | *Homo sapiens* | Thakur,S. (Direct Submission, 2007) |
| EU326228 | *L. donovani* | MHOM/KE/75/H9 | Kenya | - | Thakur,S. (Direct Submission, 2007) |
| EU326229 | *L. major* | MHOM/IL/67/JERICHO | Israel | - | Thakur,S. (Direct Submission, 2007) |
| EU683620 | *L. aethiopica* | MHOM/ET/1985/Vasa; LRC-L495 | Ethiopia | - | Nasereddin,A. (2008) |
| FJ460456 | *L. major* | MHOM/EG/06/RTC-64 | Egypt | - | Shehata,M.G. (2009) |
| FJ460459 | *L. tropica* | MHOM/EG/06/RTC-67 | Egypt | *Homo sapiens* | Shehata,M.G. (2009) |
| FN398151 | *L. lindenbergi* | MHOM/BR/1966/M15733 | Brazil | *Homo sapiens* | Kuhls,K. (Unpublished) |
| FN398152 | *L. naiffi* | MDAS/BR/1979/M5533 | Brazil | *Dasypus* sp. | Kuhls,K. (Unpublished) |
| FN398153 | *L. utingensis* | ITUB/BR/1977/M4964 | Brazil | *Lutzomyia tuberculata* | Kuhls,K. (Unpublished) |
| FN398154 | *L. lainsoni* | MHOM/BR/1981/M6426 | Brazil | *Homo sapiens* | Kuhls,K. (Unpublished) |
| FN398328 | *L. shawi* | MCEB/BR/1984/M8408 | Brazil | *Cebus apella* | Kuhls,K. (Unpublished) |
| FN398329 | *L. guyanensis* | MHOM/BR/1997/249-P | Brazil | *Homo sapiens* | Kuhls,K. (Unpublished) |
| FN398337 | *L. braziliensis* | MHOM/PE/2003/LH2920 | Peru | *Homo sapiens* | Kuhls,K. (Unpublished) |
| FN398338 | *L. braziliensis* | MHOM/BR/00/LTB300 | Brazil | *Homo sapiens* | Kuhls,K. (Unpublished) |
| FN398339 | *L. peruviana* | MHOM/PE/1990/HB86 | Peru | *Homo sapiens* | Kuhls,K. (Unpublished) |
| FN398344 | *L. donovani* | MHOM/BR/2007/JFF | Brazil | *Homo sapiens* | Kuhls,K. (Unpublished) |
| FN677358 | *L. donovani* | MHOM/SD/2007/S1 | Sudan | *Homo sapiens* | Ogado Ceasar Odiwuor,S. (2010) |
| GQ367488 | *L. infantum* | MHOM/CN/08/Jiashi-5 | China | *Homo sapiens* | Wang,J.Y. (2010) |
| GQ367489 | *L. donovani* | MHOM/CN/80/801 | China | *Homo sapiens* | Wang,J. (Unpublished) |
| GQ444144 | *L. infantum* | MHOM/IR/04/IPI-UN10 | Iran | *Homo sapiens* | Mahmoudzadeh-Niknam,H. (2010) |
| KU194936 | *L. tropica* | - | China: Nanhu Town, Hami County, Xinjiang Uygur Autonomus Region | *Eremias vermiculata* | Zhang,J.R. (2016) |
| KU194937 | *L. tropica* | - | China: Nanhu Town, Hami County, Xinjiang Uygur Autonomus Region | *Eremias vermiculata* | Zhang,J.R. (2016) |
| KU194938 | *L. tropica* | - | China: Nanhu Town, Hami County, Xinjiang Uygur Autonomus Region | *Eremias vermiculata* | Zhang,J.R. (2016) |
| KU194939 | *L.* (*Sauroleishmania*) sp. | - | China: Lukchun Town, Shanshan County, Xinjiang Uygur Autonomus Region | *Phrynocephalus axillaris* | Zhang,J.R. (2016) |
| KU194967 | *L.* (*Sauroleishmania*) sp. | - | China: Dunhuang Yardong National Geopark, Gansu Province | *Eremias vermiculata* | Zhang,J.R. (2016) |
| KU194968 | *L.* (*Sauroleishmania*) sp. | - | China: Dunhuang Yardong National Geopark, Gansu Province | *Eremias vermiculata* | Zhang,J.R. (2016) |
| KU194969 | *L.* (*Sauroleishmania*) sp. | - | China: Dunhuang Yardong National Geopark, Gansu Province | *Eremias vermiculata* | Zhang,J.R. (2016) |
| KU194970 | *L.* (*Sauroleishmania*) sp. | - | China: Dunhuang Yardong National Geopark, Gansu Province | *Eremias vermiculata* | Zhang,J.R. (2016) |
| KU194971 | *L.* (*Sauroleishmania*) sp. | - | China: Dunhuang Yardong National Geopark, Gansu Province | *Eremias vermiculata* | Zhang,J.R. (2016) |
| KU194972 | *L.* (*Sauroleishmania*) sp. | - | China: Dunhuang Yardong National Geopark, Gansu Province | *Eremias vermiculata* | Zhang,J.R. (2016) |
| KU194973 | *L.* (*Sauroleishmania*) sp. | - | China: Tuokexun County, Xinjiang Uygur Autonomus Region | *Tenuidactylus elongatus* | Zhang,J.R. (2016) |
| KU680845 | *L. major* | MHOM/SU/73/5ASKH | Russia | *Homo sapiens* | Fotouhi-Ardakani,R. (2016) |
| KU680846 | *L. major* | MRHO/IR/75/ER | Iran | *Homo sapiens* | Fotouhi-Ardakani,R. (2016) |
| KU680853 | *L. tropica* | MHOM/SU/74/K27 | Russia | *Homo sapiens* | Fotouhi-Ardakani,R. (2016) |
| KU975160 | *L. major* | MHOM/CN/2015/CPOLM-1 | China | - | Chen,Y. (Unpublished) |
| LC028235 | *L.* (*Sauroleishmania*) sp. | - | Portugal | *Sergentomyia minuta* | Maia,C. (2015) |
| LC216359 | *L.* (*Sauroleishmania*) sp. | - | Spain: Madrid | *Sergentomyia minuta* | Gonzalez,E. (Unpublished) |
| LC216360 | *L.* (*Sauroleishmania*) sp. | - | Spain: Madrid | *Sergentomyia minuta* | Gonzalez,E. (Unpublished) |
